# Response of rove‐beetle (Staphylinidae) assemblages to the cumulative effect of wildfire and linear footprint in boreal treed peatlands

**DOI:** 10.1002/ece3.9564

**Published:** 2022-12-03

**Authors:** Linhao Wu, Jaime Pinzon

**Affiliations:** ^1^ Natural Resources Canada, Canadian Forest Service Northern Forestry Center Edmonton Canada

**Keywords:** boreal Forest, Forest management, fragmentation, habitat restoration, Staphylinid biodiversity

## Abstract

Cumulative effects of anthropogenic and natural disturbances have become increasingly relevant in the context of biodiversity conservation. Oil and gas (OG) exploration and extraction activities have created thousands of kilometers of linear footprints in boreal ecosystems of Alberta, Canada. Among these disturbances, seismic lines (narrow corridors cut through the forest) are one of the most common footprints and have become a significant landscape feature influencing the maintenance of forest interior habitats and biodiversity. Wildfire is a common stand‐replacing natural disturbance in the boreal forest, and as such, it is hypothesized that its effects can mitigate the linear footprint associated with OG exploration, but only a few studies have examined its effectiveness. We studied the short‐term (1 year post‐fire) response of rove‐beetle assemblages to the combined effects of wildfire and linear footprint in forest, edge, and seismic line habitats at burned and unburned peatlands along the southwest perimeter of the 2016 Horse River wildfire (Fort McMurray). While rove‐beetle species richness was higher in seismic lines in both the burned and unburned habitats compared with the adjacent peatland, diversity was greater only in seismic lines of burned areas. Abundance was lower in the burned adjacent peatland but similarly higher in the remaining habitats. Assemblage composition on seismic lines was significantly different from that in the adjacent forest and edge habitats within both burned and unburned sites. Moreover, species composition in burned seismic lines was different from either unburned lines or burned forest and edge. *Euaesthethus laeviusculus* and *Gabrius picipennis* were indicator species of burned line habitats, are sensitive to post‐fire landscapes and can occupy wet habitats with moss cover more efficiently than when these habitats are surrounded by unburned forest. Although these results are based on short‐term responses, they suggest that wildfire did not reduce the linear footprint, and instead, the cumulative effect of these two disturbances had a more complex influence on rove‐beetle recovery at the landscape level than for other invertebrates. Therefore, continued monitoring of these sites can become useful to evaluate changes over time and to better understand longer‐term biodiversity responses to the cumulative effects of wildfire and linear disturbances in boreal treed peatlands, given the long‐lasting effect of such disturbances.

## Graphical abstract

We studied the short‐term (1 year post‐fire) responses of rove beetle assemblages to the combined effects of wildfire and linear footprint in forest, edge and seismic line habitats at burned and unburned peatlands along the southwest perimeter of the 2016 Horse River wildfire, in Fort McMurray (Alberta), with the objective to address whether fire could be used as a natural silvicultural approach to mitigating the linear footprint. The major finding of our study is that wildfire did not reduce the linear footprint, and instead, had a more complex influence on rove beetle recovery at the landscape level.

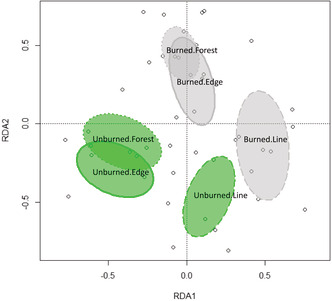

## 1 INTRODUCTION

Understanding species diversity and composition patterns in natural ecosystems and the processes driving them are of central interest in ecology (Gaston, 2000; Tscharntke et al., 2012). Disturbances, whether they are natural or anthropogenic, are a fundamental and dominant driver of change in ecosystem structure and function (McLauchlan et al., 2014; Peters et al., 2011). In many ecosystems, disturbances co‐occur over the landscape influencing both biotic and abiotic conditions that in turn drive species distributions from local to regional spatial scales (Buma, 2015; Turner, 2010). With the recent increase of resource extraction activities in much of the boreal forest of Canada and the prevalence of wildfire in this ecosystem, the cumulative effects of both anthropogenic and natural disturbances on forests have become an active area of scientific research (Fisher & Burton, 2018; Flannigan et al., 2009; Hodgson & Halpern, 2018; Thom & Seidl, 2016).

Disturbances associated with oil and gas (OG) exploration and extraction in Alberta, Canada, have become one of the most significant human footprints in the province, with the oil sands deposits covering an area of 142,000 km^2^ (Percy, 2012). Much of the oil sands deposits are too deep for conventional open‐pit mining; extending the footprint of in‐situ extraction over large areas of the province (Dabros et al., 2018; Rosa et al., 2017; Schneider, 2002). Over the last six decades, in‐situ operations have created thousands of kilometers of linear footprints including seismic lines for exploration, pipelines for distribution, and a network of access roads (Pasher et al., 2013). Seismic lines are narrow corridors cut through the forest to allow the transport and deployment of geophysical equipment to map bitumen deposits in the subsoil. This disturbance has become one of the most ubiquitous linear footprints in Alberta, and thus, an important cause of forest fragmentation (Jaeger, 2000). The presence of such disturbance is of relevance, as it poses a significant threat to maintaining forest interior habitats and biodiversity across the landscape (Dabros et al., 2018; Fisher & Burton, 2018; Pattison et al., 2016; Stern et al., 2018).

Many conventional seismic lines (10–15 m wide corridors built prior to 2000) have shown little to no natural recovery of forest cover, even after decades of construction (Jorgenson et al., 2010; Lee & Boutin, 2006; van Rensen et al., 2015). The prevalence of such linear footprint, particularly in peatlands, can persistently and severely alter soil characteristics, hydrological patterns, and permafrost (Smith, 2011; Williams et al., 2013; Zhang et al., 2000). Furthermore, it has been demonstrated that seismic lines act as corridors that facilitate the movement of wildlife across the landscape. For example, it has become the main cause of dramatic declines in woodland caribou (*Rangifer tarandus caribou*) due to increased interaction with predators, such as wolves (*Canis lupus*) (DeMars & Boutin, 2018; Dickie et al., 2017; Latham et al., 2011). The presence of seismic lines not only changed the territory delimitation of ovenbirds (*Seiurus aurocapilla*) (Bayne et al., 2005; Lankau et al., 2013), but also influenced the richness and diversity of spiders and carabid beetles (Pinzon et al., 2021) and has been shown as a refuge for butterflies (Riva et al., 2020).

Forest fragmentation is usually associated with severe ecological consequences such as biodiversity loss and degradation of ecosystem functions (Fischer & Lindenmayer, 2007; Haddad et al., 2015; Mitchell et al., 2013). Forest fragmentation reduction relies on the restoration of forest structure from the biodiversity‐ecosystem functioning perspective to ensure recovery of biodiversity and ecological processes and services (Stanturf et al., 2014). Seismic line ecological restoration approaches can be active, through direct human intervention applying different mechanical ground preparation treatments, or passive, by allowing natural disturbances to reset stand conditions (Dabros et al., 2018). Wildfire is a common stand‐replacing natural disturbance in boreal ecosystems (Bergeron et al., 2004; Reilly et al., 2006; Weber & Stocks, 1998) that is known to reset stand conditions to early successional stages (Bergeron et al., 2014). Thus, wildfire has been proposed as a natural way of mitigating the linear footprint (Dabros et al., 2017). However, prescribed burning for restoration purposes has not been widely used due to safety issues (Joshi et al., 2019), and only a few studies have examined the effectiveness of wildfire on linear footprint mitigation (Filicetti & Nielsen, 2018; Pinzon et al., 2021; Riva et al., 2020). Furthermore, there is also a large lack of understanding on how different taxa respond to the combined effect of fire over previously disturbed areas by linear features, not to mention invertebrates receiving less attention compared to vegetation or charismatic wildlife such as caribou (Dabros et al., 2018).

The use of indicator taxa has progressed as a practical method in the process of meeting ecological targets to evaluate ecosystem recovery after disturbances (Lindenmayer et al., 2000). Rove beetles (Coleoptera: Staphylinidae) are diverse and active in forest ecosystems (Brunke et al., 2019; Grimaldi & Engel, 2005; Klimaszewski et al., 2018; Thayer, 2016), do not particularly depend on the presence of mature forest but on various specific ecological niches (Thayer, 2016), and are sensitive to environmental changes (Bohac, 1999; Klimaszewski et al., 2018; Pohl et al., 2008). Consequently, rove beetles have been widely and effectively used as bioindicators to assess recovery following disturbance (Hammond et al., 2018; Klimaszewski et al., 2018; Lee et al., 2022; Nagy et al., 2016).

In this article, we study the short‐term (1 year post‐fire) responses of rove‐beetle assemblages (diversity and composition) to the combined effects of wildfire and linear footprint disturbances in forest, edge, and seismic line habitats of burned and unburned boreal treed peatlands. The work was designed to test whether wildfire serves as a silvicultural approach to mitigate the linear footprint (Dabros et al., 2017; Pinzon et al., 2021) in peatland landscapes highly fragmented by OG activities. If true, we expect assemblages of seismic lines in burned areas to be no different from those in the adjacent burned forest, but different from assemblages in both unburned seismic lines and reference unburned treed areas.

## 2 MATERIALS AND METHODS

### 2.1 Study area

**FIGURE 1:**
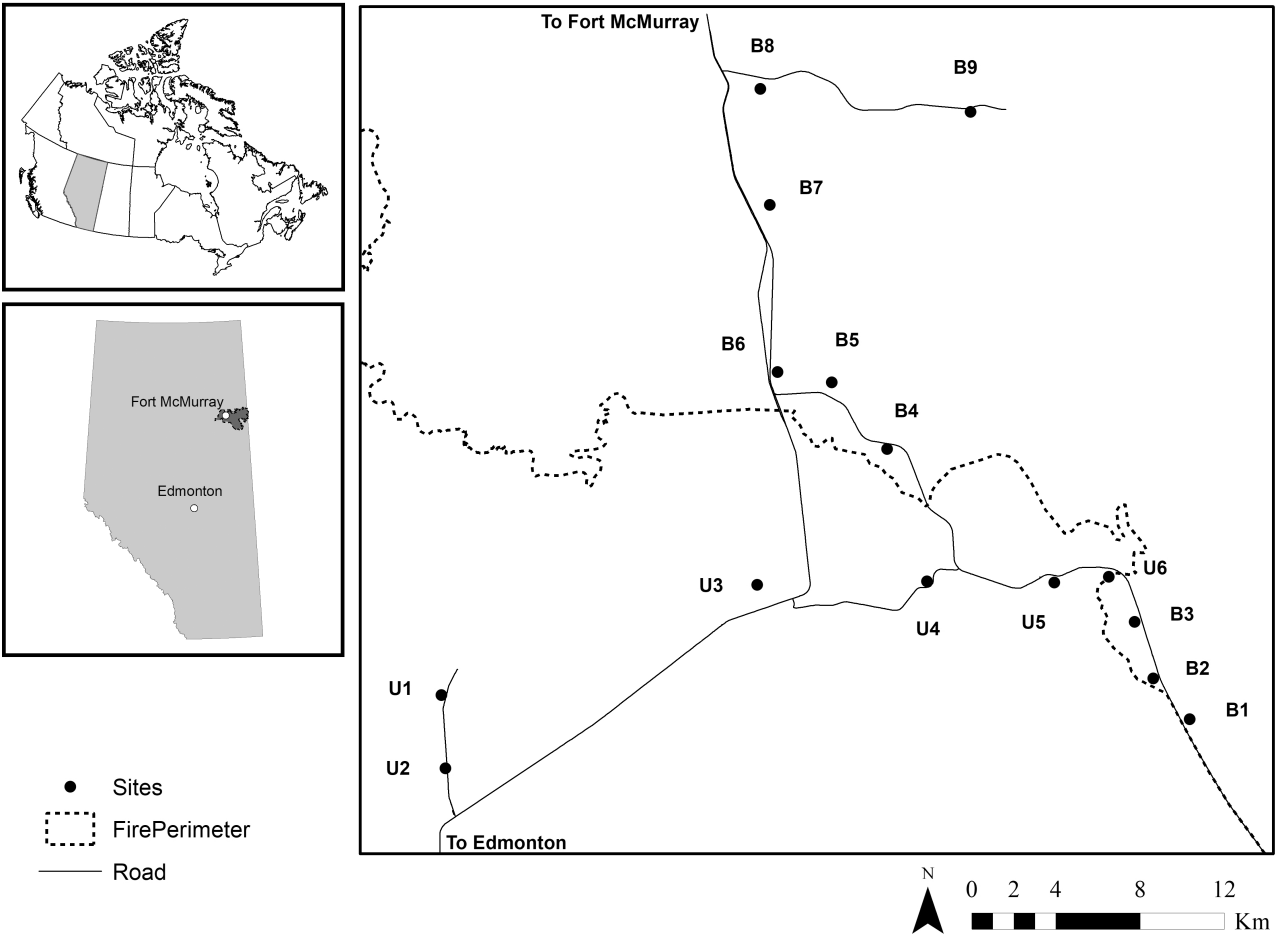
Study area along the SW perimeter of the 2016 Horse River wildfire (Fort McMurray). The location of burned (B1–B9) and unburned (U1–U6) sites are shown.

This study was conducted along the southwest perimeter of the 2016 Horse River wildfire, south of Fort McMurry, Alberta (56°46′13″ N, 118°22′28″ W; Figure 1), in the same study area as in Pinzon et al. (2021) and Riva et al. (2020). This area included 15 peatland sites within (“Burned”, *n* = 9) and outside the burned area (“Unburned”, *n* = 6). Sites were disturbed by conventional seismic lines that were built 15–20 years prior to the wildfire event. All the sites were at least 200 m from roads and were at least 2.4 km from each other. Sites were located in treed peatlands dominated by black spruce (*Picea mariana* (Miller) Britton, Sterns & Poggenburgh) in the overstory, and sphagnum (*Sphagnum* L. spp.), bog haircap (*Polytrichum stictum* Brid.), red‐stemmed feathermoss (*Pleurozium schreberi* (Brid.) Mitt.), sedges (*Carex* L. spp.), horsetails (*Equisetum* L. spp.), three‐leaved false Solomon's seal (*Maianthemum trifolium* (L.) Sloboda), Labrador tea (*Rhododendrum greoenlandicum* (Oeder) Kron & Jud), cloudberry (*Rubus chamaemorus* L.), mountain cranberry (*Vaccinium vitis‐idaea* L.), bogbirch (*Betula pumila* L.), and willows (*Salix* L. spp.) in the understory. For sites within the fire perimeter, severity of burns was low on seismic lines but severe in both forest and edge habitats. More details about the landscape in which this study took place can be found in Pinzon et al. (2021).

### 2.2 Field design and beetle sampling

At each site, we installed three parallel 50 m transects, each in one of three habitat types: along the center of the seismic line (“Line” habitat), along the forest edge approximately 10 m from the line (“Edge” habitat), and in the adjacent peatland approximately 50 m from the line (“Forest” habitat). Edge and Forest transects were located on the same side of the seismic line at each site.

We collected rove beetles using pitfall traps (1 L in volume, 12 cm diameter) dug into the peat with their upper rims leveled with the ground surface. Traps were filled with approximately 200 ml of propylene glycol as a killing agent and preservative, and were covered with a suspended opaque plastic roof to minimize flooding by rainfall and accumulation of debris (Bergeron et al., 2013; Spence & Niemelä, 1994). Along each transect, we installed five traps every 10 m for a total of 15 traps/site. We collected trap contents at 3‐week intervals between May 20 and September 15 of 2017. Adult rove beetles were sorted out from the pitfall samples in the laboratory and identified to the species level using Newton et al. (2001) and references listed therein. Specimens in the subfamily Pselaphinae were identified to the genus level since reliable taxonomic keys for local species are not available. Species in the subfamily Aleocharinae were excluded from all analyses because of difficulties in species‐level identification. Voucher specimens are deposited in the Invertebrate Museum at the Northern Forestry Center (Natural Resources Canada – Canadian Forest Service) in Edmonton, Alberta.

### 2.3 Analyses

Prior to analyses, and to account for occasional trap disturbance by wildlife, we standardized rove‐beetle catches from each trap to number of individuals per 120 trap‐days. Since the intention of installing various traps in each habitat was to account for microhabitat variability at each habitat type, we pooled the standardized catches from the five traps in each transect for all analyses. We assumed trap disturbance to have no systematic influence on the data.

As observations from transects in each habitat type are likely spatially correlated, we tested differences in standardized catch as a function of fire (Burned, Unburned) by habitat type (Line, Edge, and Forest) combinations (i.e., Fire × Habitat) using a mixed‐effects model with site as a random effect, followed by post‐hoc multiple comparisons (we have transformed the standardized abundance data by applying the square root, as with the untransformed data residuals were not normal). Since standardization only offset sampling efforts and does not account for missing species due to trap disturbance, we could not follow the above mixed‐effects model approach and instead estimated species richness and diversity (exponential of Shannon's diversity (Jost, 2006)) using coverage‐based rarefaction (Chao & Jost, 2012) for fire by habitat combinations. In the coverage‐based rarefaction, assemblages are compared with equal completeness rather than equal sampling efforts. We assessed differences in estimated values by means of 95% confidence intervals returned by the analyses, with no overlap denoting a significant difference.

We used Canonical Redundancy Analysis (RDA; Legendre & Legendre, 2012) to assess the response of rove‐beetle assemblage composition to fire and habitat combinations (i.e., Fire × Habitat), using site as a conditional variable to account for spatial correlation of transects within habitat type. Significance of the final model, explanatory variables and RDA axes were tested based on *p*‐values generated from 5000 permutations. To visually assess differences among fire by habitat groups on the ordination plots, we used ellipsoid 95% confidence intervals around group centroids.

At last, we used Indicator Species Analysis (Dufrêne & Legendre, 1997) to identify species indicative of particular fire by habitat combinations. This analysis provides indicator values (IndVal) for each species by calculating species relative abundances and relative frequencies within defined categories. Then, these values are tested against a random distribution after 4999 permutations. Species with a significant IndVal (*p*‐value <.05) are assumed to be indicators of a given category, as well as species with IndVal >0.6, are here considered strong indicators.

All analyses were performed in R v 4.1.0 (R Core Team, 2020) using the following packages: iNEXT (Hsieh et al., 2020) for coverage‐based rarefaction, nlme (Pinheiro et al., 2020) for general mixed‐effects model with least‐square mean estimation using emmeans (Lenth, 2020), vegan (Oksanen et al., 2019) for RDA analysis and indicspecies (De Cáceres & Legendre, 2009) for indicator species analysis.

## 3 RESULTS

A total of 2693 rove beetles (excluding the subfamily Aleocharinae), comprising 62 species in 12 subfamilies were collected in this study. Staphylininae (17 species) was the most species‐rich subfamily, followed by Tachyporinae (13 species) and Steninae (8 species). Tachyporinae (816 individuals) accounted for most (30.3%) of the total catch, followed by Staphylininae (25.4%, 685 individuals) and Pselaphinae (16.2%, 4 species with 486 individuals). *Reichenbachia* sp. (Casey, 1897) in the subfamily Pselaphinae was the most abundant species (14.5%, 391 individuals) followed by *Ischnosoma fimbriatum* (Campbell, 1991) in the subfamily Tachyporinae (11.0%, 297 individuals) and *Quedius frigidus* (Smetana, 1971) in the subfamily Staphylininae (9.5%, 256 individuals). Singletons (8 species) and doubletons (10 species) comprised 12.9% and 16.1% of the total richness, respectively.

### 3.1 Staphylinid richness, diversity, and abundance

**FIGURE 2:**
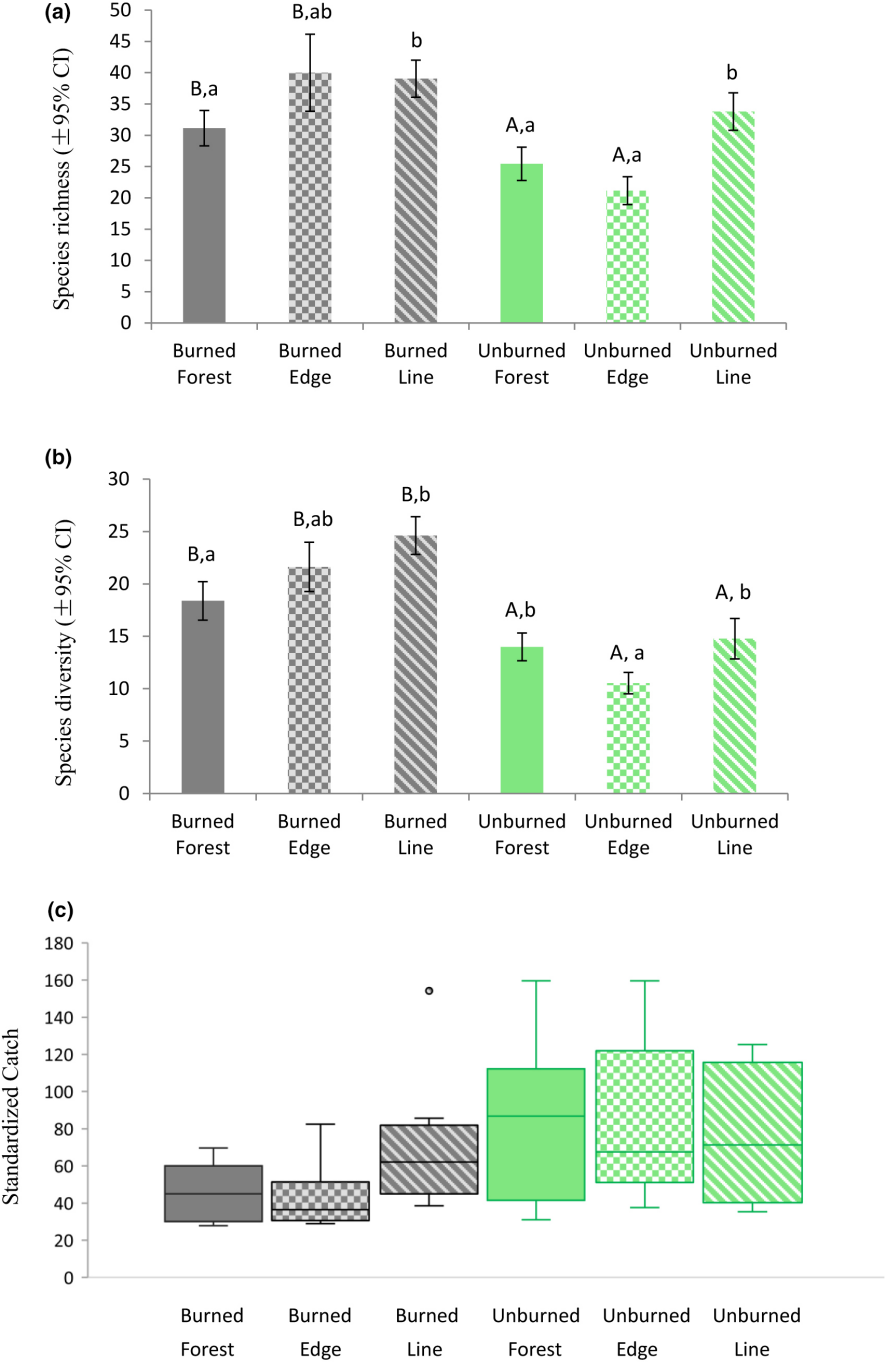
Rove beetle (Staphylinidae) species richness (a), diversity (b), and abundance (c) of in forest edge and seismic line habitats at burned and unburned peatlands along the SW perimeter of the 2016 Horse River wildfire (Fort McMurray). Different letters stand for significant differences (*α* = 0.05). Upper case letters represent differences between burned and unburned sites by habitat (e.g., burned forest vs unburned forest). Lower case letters represent differences between habitats by fire category (e.g., forest vs. edge vs. line in unburned sites). In c, the thick horizontal bars in bar plots represent the median, the boxes show interquartile range which represents the middle 50% of the data, dots are outliers, upper longitudinal bars are maxima, lower longitudinal bars are minimum.

The estimated species richness (sample coverage (SC) = 0.96) was higher in Forest and Edge habitats in burned sites compared to unburned sites. However, richness was no different between Burned and Unburned Line habitats (Figure 2a). Nonetheless, estimated richness was consistently higher in the Line habitats compared to the Forest habitats in both Burned and Unburned sites. In contrast, diversity (SC = 0.96) was significantly higher in Burned sites compared to Unburned sites, regardless of the habitat (Figure 2b). Diversity in the Line habitat was usually the highest among habitats regardless of sites being burned or not. In terms of abundance, no significant differences were detected between sites with fire category and habitat combinations (Figure 2c; Table S1).

### 3.2 Staphylinid assemblages

**FIGURE 3:**
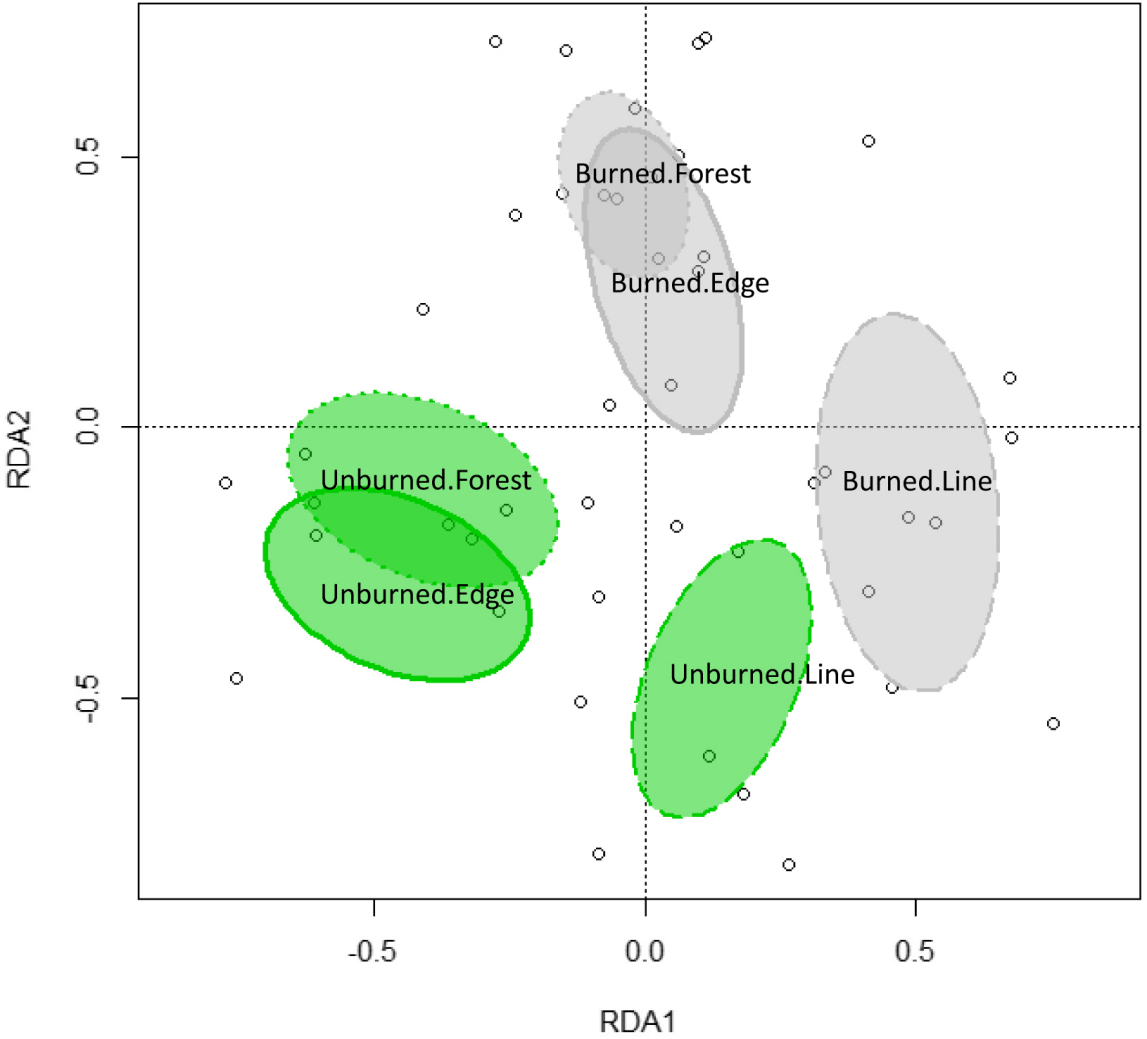
RDA ordination of rove beetle (Staphylinidae) assemblages in forest, edge, and seismic line habitats at burned and unburned peatlands along the SW perimeter of the 2016 Horse River wildfire (Fort McMurray). Ellipses are 95% confidence intervals around centroids. Green ellipses represent unburned sites and gray ellipses represent burned sites. Dotted ellipses represent forest habitats, dashed ellipses represent seismic lines and continuous ellipses represent forest edge habitats.

The RDA model was significant (*F*_5,39_ = 1.62; *p* = .001; Figure 3) though it explained only 7.0% (adjusted *R* ^2^) of the total variance, with axis 1 and axis 2, respectively, explaining 4.1% and 2.2% of the constrained variance. Nonetheless, the overall analysis supports several clear conclusions. Firstly, assemblage composition in Burned sites was significantly different from those in Unburned sites (i.e., ellipses of these sites do not overlap in the RDA ordination space, Figure 3). Second, differences in assemblage composition suggest little to no change between Forest and Edge habitat regardless of being burned or not (i.e., ellipses of these sites overlap in the RDA ordination space, Figure 3). At last, differences in species composition were observed between Burned and Unburned Line habitats as well as between Line habitats and Forest or Edge habitats in either fire category.

### 3.3 Indicator species

Seven species were identified as indicators of fire by habitat combinations (Table 1). *Euaesthethus laeviusculus* (Mannerheim, 1844) (IndVal = 0.696) and *Gabrius picipennis* (Maklin, 1852) (IndVal = 0.670) were indicator species of Burned Line sites. These two species are usually associated with wet habitats (Klimaszewski et al., 2007; Sushko, 2014). *Tachinus basalis* (Erichson, 1839) was the only indicator species of Unburned Forest with the highest indicator value (IndVal = 0.855; Table 1). This is a generalist species but prefers closed canopy (Klimaszewski et al., 2007; Lee et al., 2022). *Proteinus limbatus* (Maklin, 1852) (IndVal = 0.638), *Tachinus fumipennis* (Say, 1832) (IndVal = 0.577) and *Ischnosoma fimbriatum* (IndVal = 0.560) were indicator species of the Unburned Edge habitat. Both *P. limbatus*, *T. fumipennis* are documented as forest specialist species (Klimaszewski et al., 2007; Lee et al., 2022) and *I. fimbriatum* is a generalist species preferring open habitats and regenerating forest, but can be also found in mature forest. Only one species, *Paederus littorarius* (Gravenhorst, 1806) (IndVal = 0.678) was indicator of the Unburned Line habitat (Table 1) and has been associated with wet litter conditions (Klimaszewski et al., 2007).

**TABLE 1:** Staphylinid indicator species of forest, edge, and seismic line habitats at burned and unburned peatlands along the SW perimeter of the 2016 Horse River wildfire (Fort McMurray).

	Indicator species	Indicator value	*p‐value*
Burned Line	***Euaesthetus laeviusculus***	0.696	.001
	***Gabrius picipennis***	0.670	.009
Unburned Forest	***Tachinus basalis***	0.855	<.001
Unburned Edge	***Proteinus limbatus***	0.638	.040
	*Tachinus fumipennis*	0.577	.040
	*Ischnosoma fimbriatum*	0.560	.026
Unburned Line	***Paederus littorarius***	0.678	.003

*Note*: Strong indicators (indictor value >0.6) are highlighted in bold text.

## 4 DISCUSSION

Our results showed significant and immediate cumulative influences of seismic lines and wildfire on rove‐beetle assemblages. Fire did not fully mitigate the linear footprint, at least one year post‐disturbance, since differences in rove‐beetle richness, diversity and composition where observed between line habitats and the adjacent peatland within burned sites. This is consistent with previous studies at the same sites for butterflies, spiders, non‐vascular plants and understory plants (Pinzon et al., 2021; Riva et al., 2020) and is mainly due to the observed relatively low burn severity along the seismic lines (Pinzon et al., 2021). Seismic lines in peatlands usually exhibit little tree regeneration (Dabros et al., 2018; Lee & Boutin, 2006; Pattison et al., 2016), indirectly indicating lower fuel for the fire to burn, and are also wetter (Dabros et al., 2017; Pinzon et al., 2021; Strack et al., 2018), showing higher *Sphagnum* moss cover (Deane et al., 2020). These conditions, thus, likely prevented the lines from burning extensively, with fire skipping over and spreading through the adjacent forest. Therefore, habitat conditions in Burned Lines were more similar to those in Unburned Lines and largely different to Burned Forest and Edge habitats (Pinzon et al., 2021), which likely lead to the observed significant differences in rove‐beetle composition richness and diversity. Interestingly, despite no differences in richness and diversity with respect to edge, rove‐beetle composition was clearly different in these two habitats.

Pinzon et al. (2021) observed little difference in species composition between seismic lines within and outside the fire perimeter especially for non‐vascular plants, understory plants, and overstory plants. In contrast, differences between seismic lines in burned and unburned sites for spiders and carabid beetle composition were evident, and consistent to the rove‐beetle responses observed here. Rove‐beetle assemblages, however, showed a stronger response to seismic lines in burned and unburned sites than spiders and carabid beetles as in Pinzon et al. (2021), given no overlap in 95% confidence intervals was detected in the RDA ordination. These observations not only suggest that invertebrate assemblages seem more sensitive to fire than plants in our study area, but that rove‐beetles exhibit a much stronger response to fire despite the fact that seismic lines did not fully burn. We observed assemblage composition differences in Line habitats one year following fire, suggesting that seismic lines in burned areas are supporting different species, most of which are associated with wet habitats, according to results from the indicator species analysis. Those species seem to come from areas other than the adjacent burned forest, otherwise species composition in the Line should have been more similar to that in Burned Forest and Edge. Therefore, those species must have arrived from other areas across the landscape after the fire event and attracted to the wetter habitats that were relatively undisturbed by fire along the seismic lines. In this way, rove beetles seemed to be more sensitive to landscape level effects of burning than to local habitat conditions.

Only one species, *P. littorarius*, was associated with the Unburned Line habitats. This species is reported to prefer wet litter habitats (Klimaszewski et al., 2007) which is consistent with the wetter conditions along seismic lines. Although it was not a significant indicator for the Burned Line habitat, it was also collected in large abundances in this habitat. Two other species, *E. laeviusculus* and *G. picipennis*, were indicative of Burned Line habitats. Little is known about the biology and natural history of *E. laeviusculus* in North America, but it has been reported to be associated with wet mossy habitats and has been defined as a rare peat bog species (Sushko, 2014). As for *G. picipennis*, it is a predator and generalist species that also prefers wet mossy habitats (Klimaszewski et al., 2007). Since seismic lines were often dominated by *Sphagnum* species even after fire, therefore, it is no surprise that these two species were abundant in this habitat. Interestingly, while *E. laeviusculus* was rarely collected in unburned habitats, *G. picipennis* occurred only in burned habitats (including the adjacent burned forest); thus, the reason why they were clearly underrepresented in unburned sites remains a puzzle. Sushko (2014) collected specimes of *E. laeviusculus* by hand on *Sphagnum* hummocks and assumed they came from other sites of the bog, suggesting that this species exhibits a wide range to search for habitat and food resources. Seismic lines surrounded by burned forest created open habitats for the generalist *G. picipennis*, and these conditions may have benefited this predator to search for wet habitat covered with moss from far distances. These observations suggest these two species are sensitive to post‐fire landscape and are able to occupy wet habitats with moss cover more efficiently than when these habitats are surrounded by unburned forest.

It was surprising that no indicator species were identified to be associated with the burned forests. Several studies proved that burning of standing forest could make the forest much more attractive to many pyrophilous species (Cobb et al., 2007; Hägglund et al., 2015; Heikkala et al., 2016; Wikars, 1995). There could be several reasons to explain this observation. First, it may be due to the biology of those rove‐beetle species captured in our traps. Almost none of the rove‐beetle species in our study has been reported to be favored by fire, in contrast to other specific species of ground beetles (Cobb et al., 2007; Wikars, 1995), saproxylic beetles (rove beetles excluded) (Heikkala et al., 2016) and flat bugs (Hägglund et al., 2015) that emerged after fire. Second, one‐year post‐fire is a very short period and may not provide enough time for rove‐beetle species attracted to fire legacies in the burned forest to occupy this habitat.

Forest fragmentation usually leads to isolation of habitats and inhibits the dispersal of organisms. In our study, although wildfire did not fully mitigate the linear footprint and reduce fragmentation as we expected, it facilitated the dispersal of several species across the landscape towards relatively unburned seismic lines. However, it is still too early in the post‐fire recovery of this landscape to conclude whether such dispersal is ecologically beneficial for maintaining biodiversity in burned landscapes previously fragmented by linear disturbances. Also, as the effects of fragmentation are strong and markedly long lasting (Haddad et al., 2015), it remains unknown how long such landscape level species dispersal could last.

In conclusion, based on the rove‐beetle responses, our study restates that the effectiveness of wildfire to mitigate the linear footprint associated with OG exploration in peatlands is limited in the short term, which is consistent with previous studies addressing the same research question with other taxa (Pinzon et al., 2021; Riva et al., 2020). Thus, wildfire may not be an effective silvicultural approach, at least in peatlands, to mitigate such linear footprint. Low‐fire severity along seismic lines has important implications for post‐fire restoration but differs among taxa. Riva et al. (2020) suggest that seismic lines may serve as habitat refuge for some butterflies while Pinzon et al. (2021) argue that it may further increase the effects of linear footprint for other taxa. Interestingly, the species composition of rove beetles in burned seismic lines was neither similar to unburned line nor to burned forest, which suggests a more complex influence of fire on previously fragmented landscapes with respect to rove‐beetle recovery trajectories. Therefore, longer‐term ecosystem‐based monitoring on different taxa is crucial to understand how boreal treed peatlands respond to the cumulative effect of wildfire and linear disturbances, to better inform potential restoration efforts of these habitats.

## AUTHOR CONTRIBUTIONS

**Linhao Wu:** Formal analysis (lead); writing – original draft (lead); writing – review and editing (equal). **Jaime Pinzon:** Conceptualization (lead); investigation (lead); methodology (lead); writing – review and editing (equal).

## Supporting information


Table S1.

## Data Availability

Data are available from the Dryad Digital Repository (Pinzon & Wu, 2022; https://doi.org/10.5061/dryad.xd2547dmj.

## References

[ece39564-bib-0001] Bayne, E. M. , Boutin, S. , Tracz, B. , & Charest, K. (2005). Functional and numerical responses of ovenbirds (*Seiurus aurocapilla*) to changing seismic exploration practices in Alberta's boreal forest. Ecoscience, 12, 216–222.

[ece39564-bib-0002] Bergeron, J. A. C. , Spence, J. R. , Volney, W. J. A. , Pinzon, J. , & Hartley, D. J. (2013). Effect of habitat type and pitfall trap installation on captures of eipgaeic arthropod assemblages in the boreal forest. The Canadian Entomologist, 145, 547–565.

[ece39564-bib-0003] Bergeron, Y. , Chen, H. Y. H. , Kenkel, N. C. , Leduc, A. L. , & Macdonald, S. E. (2014). Boreal mixedwood stand dynamics: Ecological processes underlying multiple pathways. The Forestry Chronicle, 90, 202–213.

[ece39564-bib-0004] Bergeron, Y. , Flannigan, M. , Gauthier, S. , Leduc, A. , & Lefort, P. (2004). Past, current and future fire frequency in the Canadian boreal forest: Implications for sustainable forest management. Ambio, 33, 356–360. 15387074 10.1579/0044-7447-33.6.356

[ece39564-bib-0005] Bohac, J. (1999). Staphylinid beetles as bioindicators. Agriculture, Ecosystems and Environment, 74, 357–372.

[ece39564-bib-0006] Brunke, A. J. , Bouchard, P. , Douglas, H. B. , & Pentinsaari, M. (2019). Coleoptera of Canada. In D. W. Langor & C. S. Sheffield (Eds.) the biota of Canada – A biodiversity assessment. Part 1: The terrestrial arthropods. ZooKeys, 819, 361–376. 10.3897/zookeys.819.24724 PMC6355730 30713451

[ece39564-bib-0007] Buma, B. (2015). Disturbance interactions: Characterization, prediction, and the potential for cascading effects. Ecosphere, 6, 1–15.

[ece39564-bib-0008] Chao, A. , & Jost, L. (2012). Coverage‐based rarefaction and extrapolation: Standardizing samples by completeness rather than size. Ecology, 93(12), 2533–2547. 23431585 10.1890/11-1952.1

[ece39564-bib-0009] Cobb, T. P. , Langor, D. W. , & Spence, J. R. (2007). Biodiversity and multiple disturbances: Boreal forest ground beetle (Coleoptera: Carabidae) responses to wildfire, harvesting, and herbicide. Canadian Journal of Forest Research, 37, 1310–1323.

[ece39564-bib-0010] Dabros, A. , James Hammond, H. E. , Pizon, J. , Pinno, B. , & Langor, D. (2017). Edge influence of low‐impact seismic lines for oil exploration on upland forest vegetation in northern Alberta (Canada). Forest Ecology and Management, 400, 278–288.

[ece39564-bib-0011] Dabros, A. , Pyper, M. , & Castilla, G. (2018). Seismic lines in the boreal and arctic ecosystems of North America: Environmental impacts, challenges, and opportunities. Environmental Reviews, 26, 214–229.

[ece39564-bib-0012] De Cáceres, M. , & Legendre, P. (2009). Associations between species and groups of sites: Indices and statistical inference. Ecology, 90, 3566–3574. 20120823 10.1890/08-1823.1

[ece39564-bib-0013] Deane, P. J. , Wilkinson, S. L. , Moore, P. A. , & Waddington, J. M. (2020). Seismic lines in treed boreal peatlands and analogs for wildfire fuel modification treatments. Firehouse, 3, 21.

[ece39564-bib-0014] DeMars, C. A. , & Boutin, S. (2018). Nowhere to hide: Effects of linear features on predator‐prey dynamics in a large mammal system. Journal of Animal Ecology, 87, 274–284. 28940254 10.1111/1365-2656.12760

[ece39564-bib-0015] Dickie, M. , Serrouya, R. , McNay, R. S. , & Boutin, S. (2017). Faster and farther: Wolf movement on linear features and implications for hunting behaviour. Journal of Applied Ecology, 54, 253–263.

[ece39564-bib-0016] Dufrêne, M. , & Legendre, P. (1997). Species assemblages and indicator species: The need for a flexible asymmetrical approach. Ecological Monographs, 67, 345–366.

[ece39564-bib-0017] Filicetti, A. T. , & Nielsen, S. E. (2018). Fire and forest recovery on seismic lines in sandy upland jack pine (*Pinus banksiana*) forests. Forest Ecology and Management, 421, 32–39.

[ece39564-bib-0018] Fischer, J. , & Lindenmayer, D. B. (2007). Landscape modification and habitat fragmentation: A synthesis. Global Ecology and Biogeography, 16, 265–280.

[ece39564-bib-0019] Fisher, J. T. , & Burton, A. C. (2018). Wildfire winners and losers in an oil sands landscape. Frontiers in Ecology and the Environment, 16, 323–328.

[ece39564-bib-0020] Flannigan, M. D. , Krawchuk, M. A. , de Groot, W. J. , Wotton, M. B. , & Gowman, L. M. (2009). Implications of changing climate for global wildland fire. International Journal of Wildland Fire, 18, 483–507.

[ece39564-bib-0021] Gaston, K. J. (2000). Global patterns in biodiversity. Nature, 405(6783), 220–227. 10821282 10.1038/35012228

[ece39564-bib-0022] Grimaldi, D. , & Engel, M. S. (2005). Evolution of the insects. Cambridge University Press.

[ece39564-bib-0023] Haddad, N. M. , Brudvig, L. A. , Clobert, J. , Davies, K. F. , Gonzalez, A. , Holt, R. D. , Lovejoy, T. E. , Sexton, J. O. , Austin, M. P. , Collins, C. D. , Cook, W. M. , Damschen, E. I. , Ewers, R. M. , Foster, B. L. , Jenkins, C. N. , King, A. J. , Laurance, W. F. , Levey, D. J. , Margules, C. R. , … Townshend, J. R. (2015). Habitat fragmentation and its lasting impact on Earth's ecosystems. Science Advances, 1, e1500052. 26601154 10.1126/sciadv.1500052 PMC4643828

[ece39564-bib-0024] Hägglund, R. , Hekkala, A. M. , Hjälten, J. , & Tolvanen, A. (2015). Positive effects of ecological restoration on rare and threatened flat bugs (Heteroptera: Aradidae). Journal of Insect Conservation, 19, 1089–1099.

[ece39564-bib-0025] Hammond, H. E. J. , Hoffman, P. G. K. , Pinno, B. D. , Pinzon, J. , Klimaszewski, J. , & Hartley, D. J. (2018). Response of ground and ***s (Coleoptera: Carabidae, Staphylinidae) to operational oil sands mine reclamation in northeastern Alberta, a case study. Journal of Insect Conservation, 22, 687–706.

[ece39564-bib-0026] Heikkala, O. , Martikainen, P. , & Kouki, J. (2016). Decadal effects of emulating natural disturbances in forest management on saproxylic beetle assemblages. Biological Conservation, 194, 39–47.

[ece39564-bib-0027] Hodgson, E. E. , & Halpern, B. S. (2018). Investigating cumulative effects across ecological scales. Conservation Biology, 33, 22–32. 29722069 10.1111/cobi.13125

[ece39564-bib-0028] Hsieh, T. C. , Ma, K. H. , & Chao, A. (2020). iNEXT: iNterpolation and EXTrapolation for species diversity. R Package.

[ece39564-bib-0029] Jaeger, J. A. G. (2000). Landscape division, splitting index, and effective mesh size: New measures of landscape fragmentation. Landscape Ecology, 15, 115–130.

[ece39564-bib-0030] Jorgenson, J. C. , Hoef, J. M. V. , & Jorgenson, M. (2010). Long‐term recovery patterns of arctic tundra after winter seismic exploration. Ecological Applications, 20, 205–221. 20349841 10.1890/08-1856.1

[ece39564-bib-0031] Joshi, O. , Poudyal, N. C. , Weir, J. R. , Fuhlendorf, S. D. , & Ochuodho, T. O. (2019). Determinants of perceived risk and liability concerns associated with prescribed burning in the United States. Journal of Environmental Management, 230, 379–385. 30293022 10.1016/j.jenvman.2018.09.089

[ece39564-bib-0032] Jost, L. (2006). Entropy and diversity. Oikos, 113(2), 363–375.

[ece39564-bib-0033] Klimaszewski, J. , Brunke, A. J. , Work, T. T. , & Venier, L. (2018). Rove beetles (Coleoptera, *Staphylinidae*) as bioindicators of change in boreal forests and their biological control services in agroecosystems: Canadian case studies. In O. Betz , U. Irmler , & J. Klimaszewski (Eds.), Biology of rove beetles (Staphylinidae): Life history, evolution, ecology and distribution (pp. 161–181). Springer.

[ece39564-bib-0034] Klimaszewski, J. , Langor, D. W. , Savard, K. , Pelletier, G. , Chandler, D. S. , & Sweeney, J. (2007). Rove beetles (Coleoptera: Staphylinidae) in yellow birch‐dominated stands of southeastern Quebec, Canada: Diversity, abundance, and description of a new species. The Canadian Entomologist, 139, 793–833.

[ece39564-bib-0035] Lankau, H. , Bayne, E. , & Machtans, C. (2013). Overnbird (*Seiurus aurocapilla*) territory placement near seismic lines s influenced by forest regeneration and conspecific density. Avian Conservation Ecology, 8(1), 5.

[ece39564-bib-0036] Latham, A. D. M. , Latham, M. C. , Boyce, M. S. , & Boutin, S. (2011). Movement responses by wolves to industrial linear features and their effect on woodland caribou in northeastern Alberta. Ecological Applications, 21(8), 2854–2856.

[ece39564-bib-0037] Lee, P. , & Boutin, S. (2006). Persistence and developmental transition of wide seismic lines in the western Boreal Plains of Canada. Journal of Environmental Management, 78, 240–250. 16112795 10.1016/j.jenvman.2005.03.016

[ece39564-bib-0038] Lee, S. , Langor, D. W. , Pinzon, J. , Pohl, G. R. , Spence, J. R. , Hartley, D. J. , Work, T. T. , & Wu, L. (2022). Rapid recovery of rove beetle (Staphylinidae) assemblages 16 year after variable retention harvest. Ecography. 10.1111/ecog.06347

[ece39564-bib-0039] Legendre, P. , & Legendre, L. (2012). Numerical Ecology (3rd English ed.). Ellsevier.

[ece39564-bib-0040] Lenth, R. (2020). emmeans: Estimated Marginal Means, aka Least‐Squares Means. R Package.

[ece39564-bib-0041] Lindenmayer, D. B. , Margules, C. , & Botkin, D. (2000). Indicators of biodiversity for ecologically sustainable forest management. Conservation Biology, 14, 941–950.

[ece39564-bib-0042] McLauchlan, K. K. , Higuera, P. E. , Gavin, D. G. , Perakis, S. S. , Mack, M. C. , Alexander, H. , Battles, J. , Biondi, F. , Buma, B. , Colombaroli, D. , Enders, S. K. , Engstrom, D. R. , Hu, F. S. , Marlon, J. R. , Marshall, J. , McGlone, M. , Morris, J. L. , Nave, L. E. , Shuman, B. , … Williams, J. J. (2014). Reconstructing disturbances and their biogeochemical consequences over multiple timescales. Bioscience, 64(2), 105–116.

[ece39564-bib-0043] Mitchell, M. G. E. , Bennett, E. M. , & Gonzalez, A. (2013). Linking landscape connectivity and ecosystem service provision: Current knowledge and research gaps. Ecosystems, 16, 894–908.

[ece39564-bib-0044] Nagy, D. D. , Magura, T. , Mizser, S. , Debnár, Z. , & Tóthmérész, B. (2016). Recovery of surface‐dwelling assemblages (Coleoptera: Carabidae, Staphylinidae) during clear‐cut originated reforestation with native tree species. Periodicum Biologorum, 118(3), 195–203.

[ece39564-bib-0045] Newton, A. F. , Thayer, M. K. , Ashe, J. S. , & Chandler, D. S. (2001). 22. Staphylinidae Latreille, 1802. In R. H. J. Arnett & M. C. Thomas (Eds.), American beetles. Volume 1: Archostemata, Myxophaga, Adephaga, Polyphaga: Staphyliniformia (pp. 272–418). CRC Press.

[ece39564-bib-0046] Oksanen, J. , Blanchet, F. G. , Friendly, M. , Kindt, R. , Lagendre, P. , McGlinn, D. , Minchin, P. R. , O'Hara, B. , Simpson, G. L. , Solymos, P. , Stevens, M. H. H. , Szoecs, E. , & Wagner, H. (2019). vegan: Community Ecology package. R Package.

[ece39564-bib-0047] Pasher, J. , Seed, E. , & Duffe, J. (2013). Development of boreal ecosystem anthropogenic disturbance layers for Canada based on 2008 to 2010 Landsat imagery. Canadian Journal of Remote Sensing, 39, 42–58.

[ece39564-bib-0048] Pattison, C. A. , Quinn, M. S. , Dale, P. , & Catterall, C. P. (2016). The landscape impact of linear seismic clearing for oil and gas development in boreal Forest. Northwest Science, 90, 340–354.

[ece39564-bib-0049] Percy, K. E. (2012). Alberta oil sands: Energy, industry and the environment. Elsevier.

[ece39564-bib-0050] Peters, D. P. , Lugo, A. E. , Chapin, F. S., III , Pickett, S. T. , Duniway, M. , Rocha, A. V. , Swanson, F. J. , Laney, C. , & Jones, J. (2011). Cross‐system comparisons elucidate disturbance complexities and generalities. Ecosphere, 2, 81.

[ece39564-bib-0051] Pinheiro, J. , Bates, D. , DebRoy, S. , Sarkar, D. , & Team, R. C. (2020). nlme: Linear and Nonlinear mixed effects Models. R Package.

[ece39564-bib-0052] Pinzon, J. , Dabros, A. , Riva, F. , & Glasier, J. R. N. (2021). Short‐term effects of wildfire in boreal peatlands: Does fire mitigate the linear footprint of oil and gas exploration. Ecological Applications, 31(3), e02281. 33336476 10.1002/eap.2281 PMC8047916

[ece39564-bib-0053] Pinzon, J. , & Wu, L. (2022). Rove beetle (Staphylinidae) assemblages following the cumulative effect of wildfire and linear footprint in Boreal treed peatlands of northeastern Alberta (Canada). Dryad, Dataset. 10.5061/dryad.xd2547dmj PMC9719082 36479034

[ece39564-bib-0055] Pohl, G. R. , Langor, D. W. , Klimaszewski, J. , Work, T. T. , & Paquin, P. (2008). Rove beetles (Coleoptera: Staphylinidae) in northern Neartic forests. The Canadian Entomologist, 140, 415–436.

[ece39564-bib-0054] R Core Team . (2020). R: A language and environment for statistical computing. R Foundation for Statistical Computing.

[ece39564-bib-0056] Reilly, M. J. , Wimberly, M. C. , & Newell, C. L. (2006). Wildfire effects on plant species richness at multiple spatial scales in forest communities of the southern Appalachians. Journal of Ecology, 94, 118–130.

[ece39564-bib-0057] Riva, F. , Pinzon, J. , Acorn, J. H. , & Nielsen, S. E. (2020). Composite effects of Cutlines and wildfire result in fire refuges for plants and butterflies in boreal treed peatlands. Ecosystems, 23, 485–497.

[ece39564-bib-0058] Rosa, L. , Davis, K. F. , Rulli, M. C. , & D' Odorico, P. (2017). Environmental consequences of oil production from oil sands. Earth's Future, 5, 158–170.

[ece39564-bib-0059] Schneider, R. R. (2002). Alternative futures: Alberta's boreal forest at the crossroads. Federation of Alberta Naturalists Edmonton.

[ece39564-bib-0060] Smith, S. (2011). Trends in permafrost conditions and ecology in northern Canada. Canadian biodiversity: ecosystem status and trends 2010. Canadian Councils of Resource Ministers.

[ece39564-bib-0061] Spence, J. R. , & Niemelä, J. K. (1994). Sampling carabid assemblages with pitfall traps—The madness and the method. The Canadian Entomologist, 126, 881–894.

[ece39564-bib-0062] Stanturf, J. A. , Palik, B. J. , & Dumroese, R. K. (2014). Contemporary forest restoration: A review emphasizing function. Forest Ecology and Management, 331, 292–323.

[ece39564-bib-0063] Stern, E. R. , Riva, F. , & Nielsen, S. E. (2018). Effects of narrow linear disturbances on light and wind patterns in fragmented boreal forests in northeastern Alberta. Forests, 9, 486.

[ece39564-bib-0064] Strack, M. , Softa, D. , Bird, M. , & Xu, B. (2018). Impact of winter roads on boreal peatland carbon exchange. Global Change Biology, 24(1), e201–e212. 28755391 10.1111/gcb.13844

[ece39564-bib-0065] Sushko, G. G. (2014). Spatial distribution of epigeic beetles (Insecta, Coleoptera) in the “Yelnia” peat bog. Baltic Journal of Coleopterology, 14(2), 151–161.

[ece39564-bib-0066] Thayer, M. K. (2016). Staphylinidae Latreille, 1802. In R. G. Beutel & R. A. B. Leschen (Eds.), Handbook of zoology. Coleoptera, beetles – volume 1: Morphology and systematics (Archostemata, Adephaga, Myxophaga, Polyphaga partim) (2nd ed., pp. 394–442). Walter de Gruyter GmbH.

[ece39564-bib-0067] Thom, D. , & Seidl, R. (2016). Natural disturbance impacts on ecosystem services and biodiversity in temperate and boreal forests. Biological Reviews of the Cambridge Philosophical Society, 91, 760–781. 26010526 10.1111/brv.12193 PMC4898621

[ece39564-bib-0068] Tscharntke, T. , Tylianakis, J. M. , Rand, T. R. , Didham, R. K. , Fahrig, L. , Batary, P. , Bengtsson, J. , Clough, Y. , Crist, T. C. , Dormann, C. F. , Ewers, R. M. , Frund, J. , Holt, R. D. , Holzschuh, A. , Klein, A. M. , Kleijin, D. , Kremen, C. , Landis, D. A. , Laurance, W. , … Westphal, C. (2012). Landscape moderation of biodiversity patterns and processes – Eight hypotheses. Biological Reviews, 87(3), 661–685. 22272640 10.1111/j.1469-185X.2011.00216.x

[ece39564-bib-0069] Turner, M. G. (2010). Disturbance and landscape dynamics in a changing world. Ecology, 91, 2833–2849. 21058545 10.1890/10-0097.1

[ece39564-bib-0070] van Rensen, C. K. , Nielsen, S. E. , White, B. , Vinge, T. , & Lieffers, V. J. (2015). Natural regeneration of forest vegetation on legacy seismic lines in boreal habitats in Alberta's oil sands region. Biological Conservation, 184, 127–135.

[ece39564-bib-0071] Weber, M. G. , & Stocks, B. J. (1998). Forest fires and sustainability in the boreal forests of Canada. Ambio, 27, 545–550.

[ece39564-bib-0072] Wikars, L. O. (1995). Clear‐cutting before burning prevents establishment of the fire‐adapted Agonum quadripunctatum (Coleoptera: Carabidae). Annales Zoologici Fennici, 32, 375–384.

[ece39564-bib-0073] Williams, T. J. , Quinton, W. L. , & Baltzer, J. L. (2013). Linear disturbance on discontinuous permafrost: Implications for thaw‐induced changes to land cover and drainage patterns. Environmental Research Letters, 8(2), 025006.

[ece39564-bib-0074] Zhang, X. , Vincent, L. A. , Hogg, W. D. , & Niitsoo, A. (2000). Temperature and precipitation trends in Canada during the 20^th^ century. Atmosphere‐Ocean, 38(3), 395–429.

