# Supplementary material for: Response of rove‐beetle (Staphylinidae) assemblages to the cumulative effect of wildfire and linear footprint in boreal treed peatlands
Source: Ecol Evol. 2022 Dec 3;12(12):e9564. doi: 10.1002/ece3.9564 (PMC9719082; doi:10.1002/ece3.9564)
Supplement: Supplementary file 1 — Table S1. [file ECE3-12-e9564-s001.docx]

**Appendix**

**Table 1.** Pair comparisons results from mixed effects model for standardized catch of rove beetles (Staphylinidae) of forest, edge and seismic line habitats at burned and unburned peatlands along the SW perimeter of the 2016 Horse River wildfire (Fort McMurray).

| **Contrast** | **estimate** | **SE** | **t.ratio** | ***p*** |
| --- | --- | --- | --- | --- |
| Burned Edge-Burned Forest | -1.67 | 15.3 | -0.109 | 1.0000 |
| Burned Edge-Burned Line | -27.02 | 15.3 | -1.763 | 0.5001 |
| Burned Edge-Unburned Edge | -39.64 | 17.1 | -2.314 | 0.2131 |
| Burned Edge-Unburned Forest | -41.07 | 17.1 | -2.397 | 0.1823 |
| Burned Edge-Unburned Line | -33.04 | 17.1 | -1.928 | 0.4010 |
| Burned Forest-Burned Line | -25.36 | 15.3 | -1.655 | 0.5687 |
| Burned Forest-Unburned Edge | -37.98 | 17.1 | -2.217 | 0.2536 |
| Burned Forest-Unburned Forest | -39.40 | 17.1 | -2.300 | 0.2186 |
| Burned Forest-Unburned Line | -31.37 | 17.1 | -1.831 | 0.4586 |
| Burned Line-Unburned Edge | -12.62 | 17.1 | -0.737 | 0.9761 |
| Burned Line-Unburned Forest | -14.05 | 17.1 | -0.820 | 0.9621 |
| Burned Line-Unburned Line | -6.01 | 17.1 | -0.351 | 0.9992 |
| Unburned Edge-Unburned Forest | -1.43 | 18.8 | -0.076 | 1.0000 |
| Unburned Edge-Unburned Line | 6.61 | 18.8 | 0.352 | 0.9992 |
| Unburned Forest-Unburned Line | 8.04 | 18.8 | 0.428 | 0.9980 |
